# Supplementary material for: Capture of single Ag atoms through high-temperature-induced crystal plane reconstruction
Source: Nat Commun. 2024 May 8;15:3874. doi: 10.1038/s41467-024-47836-x (PMC11078991; doi:10.1038/s41467-024-47836-x)
Supplement: Supplementary file 1 — Supplementary Information [file 41467_2024_47836_MOESM1_ESM.pdf]

Supplementary Information

Capture of Single Ag Atoms through High-Temperature-Induced Crystal Plane Reconstruction

Jiaxin Li[1,†], Kai Li[1,2,†], Zhao Li[1], Chunxue Wang[1], Yifei Liang[1], Yatong Pang[1], Jinzhu Ma[3,4,5,*], Fei Wang[1,2,*], Ping Ning[1,2], Hong He[3,4,5]

[1]Faculty of Environmental Science and Engineering, Kunming University of Science and Technology, Kunming, 650500, China

[2]National-Regional Engineering Center for Recovery of Waste Gases from Metallurgical and Chemical Industries, Kunming, 650500, China

[3]State Key Joint Laboratory of Environment Simulation and Pollution Control, Research Center for Eco-Environmental Sciences, Chinese Academy of Sciences, Beijing, 100085 China

[4]Center for Excellence in Regional Atmospheric Environment, Institute of Urban Environment, Chinese Academy of Sciences, Xiamen, 361021 China

[5]University of Chinese Academy of Sciences, Beijing 100049, China

[†]These authors contributed equally: Jiaxin Li, Kai Li

*Email: jzma@rcees.ac.cn, wangfei@kust.edu

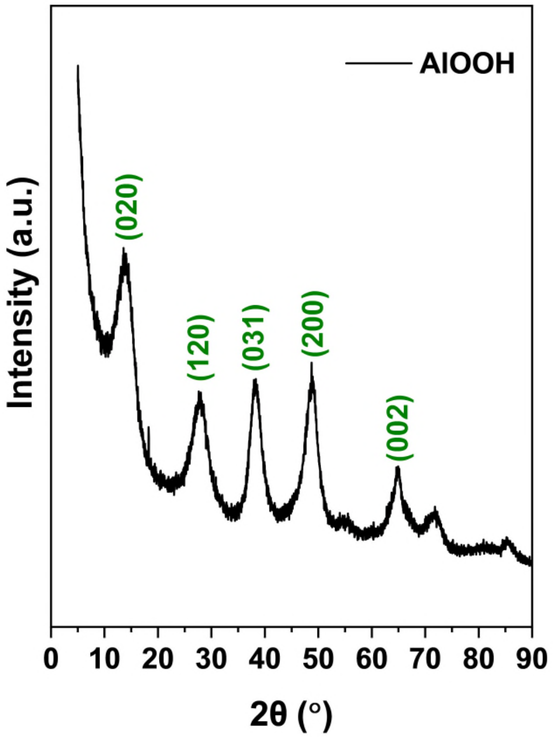

Supplementary Figure 1. XRD profiles of fresh AlOOH.

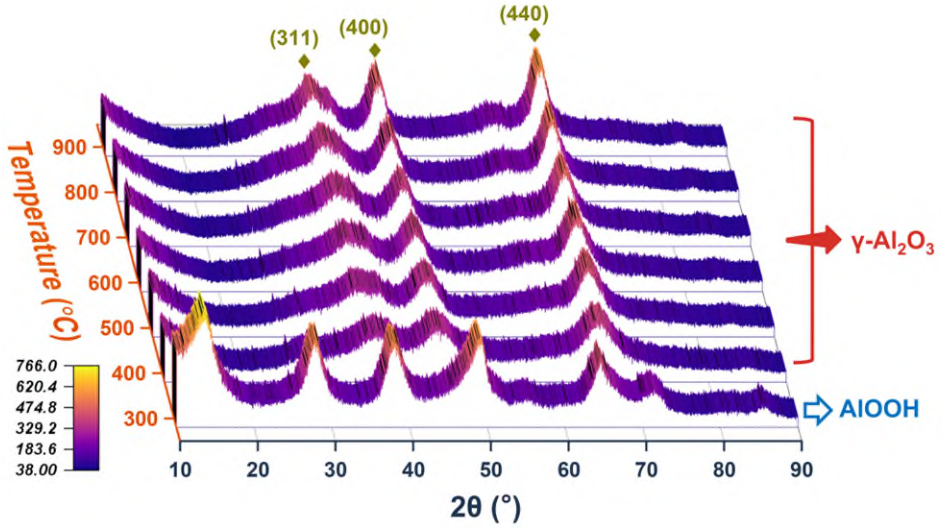

Supplementary Figure 2. In situ XRD profiles of AlOOH calcined at different temperatures.

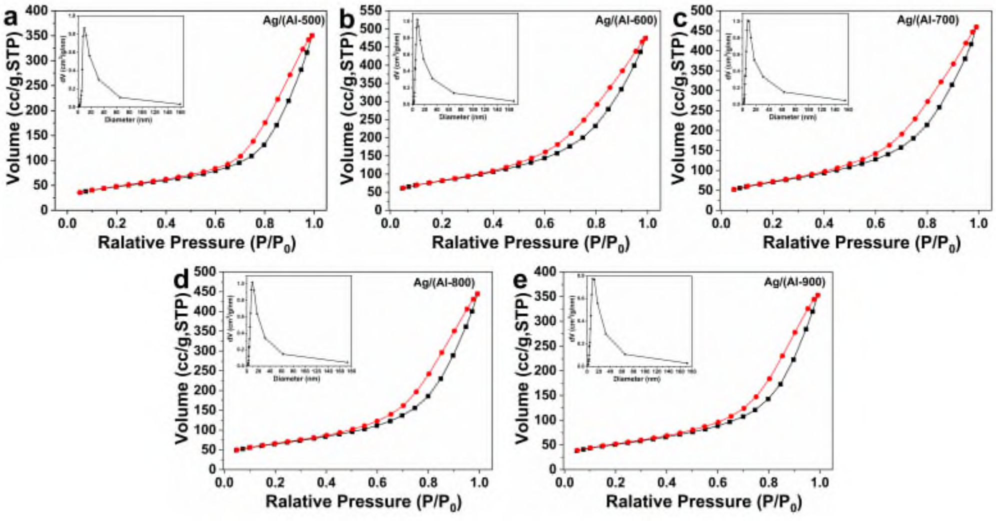

Supplementary Figure 3. N_2 adsorption-desorption isotherms and pore-size distribution curves (insets) of the Ag/(Al-X) samples. **a** Ag/(Al-500). **b** Ag/(Al-600). **c** Ag/(Al-700). **d** Ag/(Al-800). **e** Ag/(Al-900).

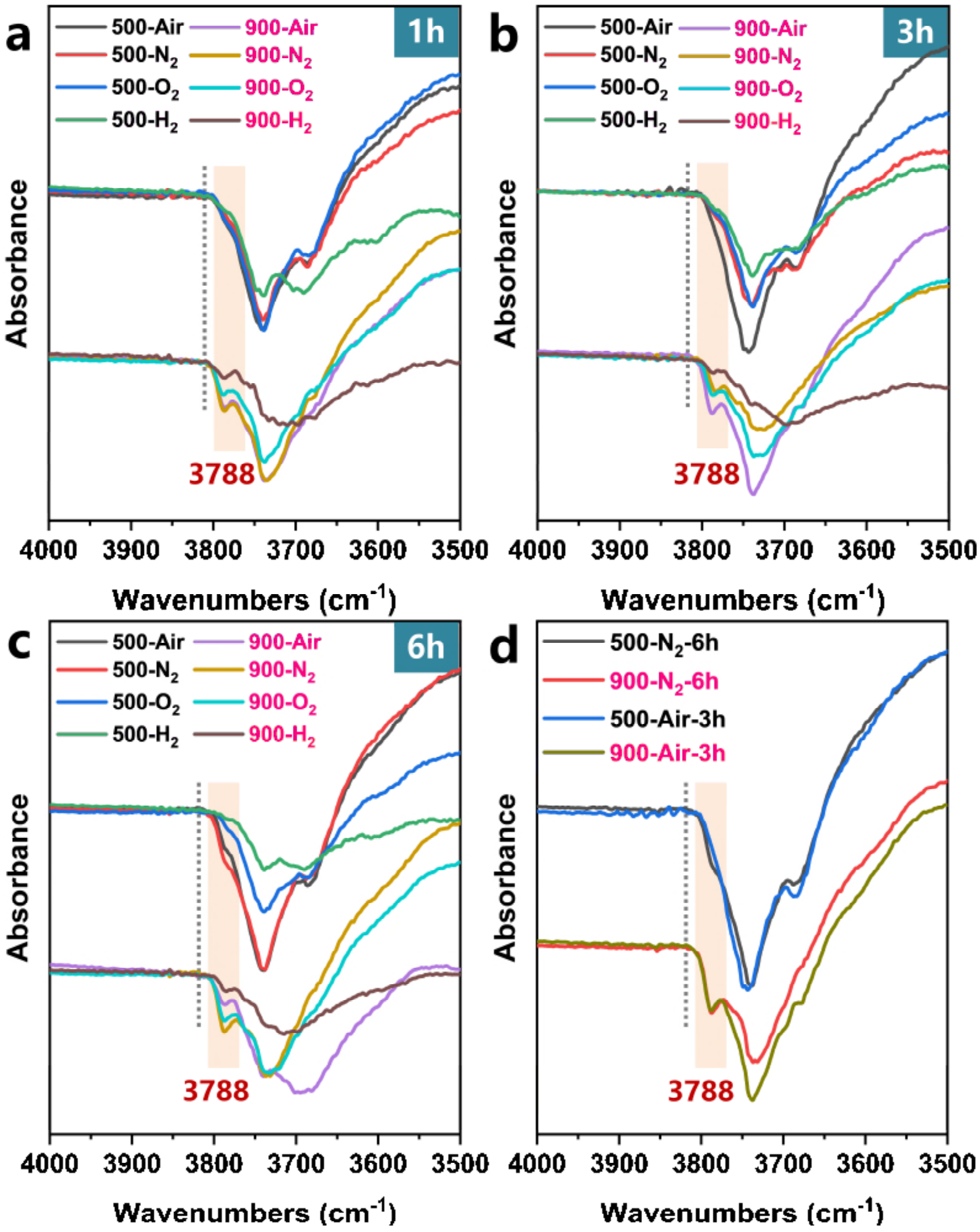

Supplementary Figure 4. Hydroxyl changes under different calcination atmospheres and time at 500 and 900 °C calcination temperature. a 1h. **b** 3h. **c** 6h. **d** Screening for optimal conditions at 500 and 900 °C calcination temperature.

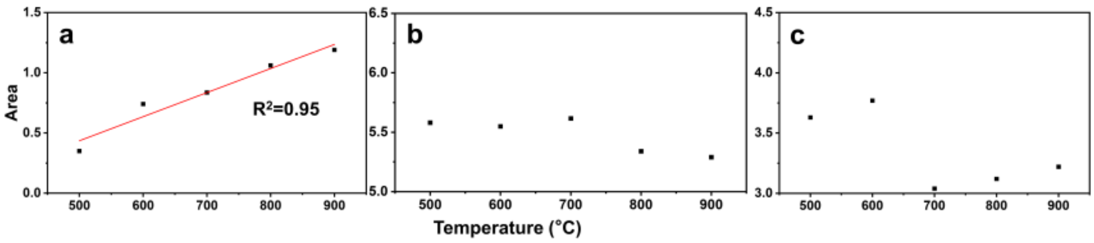

Supplementary Figure 5. Relationship between calcination temperature and peak areas of hydroxyl groups. a terminal-OH. **b** doubly-OH. **c** triply-OH.

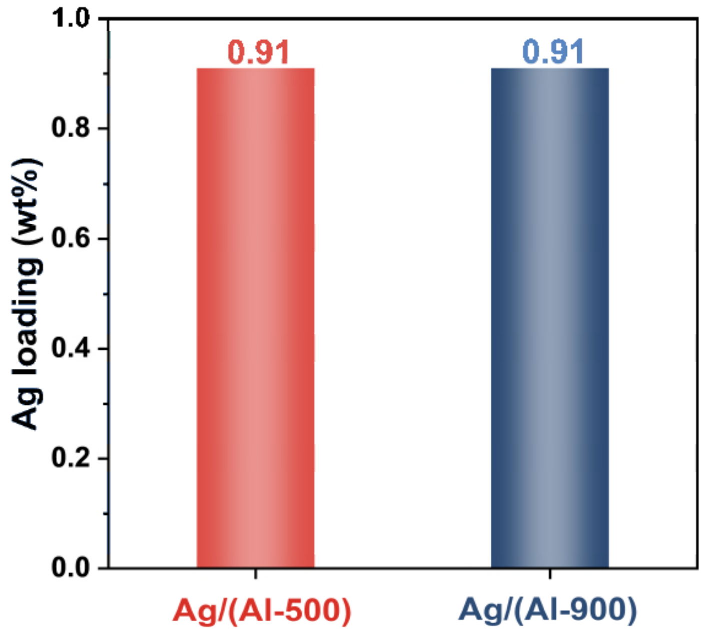

Supplementary Figure 6. ICP measurement of Ag content of Ag/(Al-500) and Ag/(Al-900) samples.

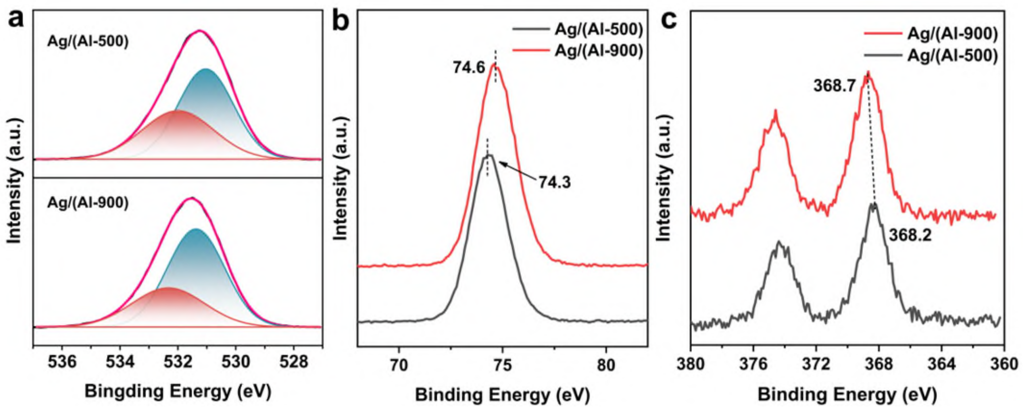

Supplementary Figure 7. XPS spectra of Ag/(Al-500) and Ag/(Al-900) samples. a O 1*s*. **b** Al 2*p*. **c** Ag 3*d*.

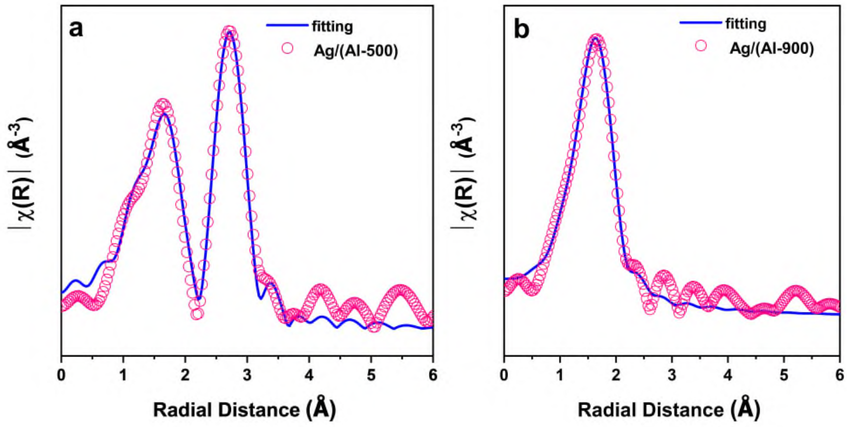

Supplementary Figure 8. EXAFS fitting curve in R space. a Ag/(Al-500). **b** (Ag/Al-900).

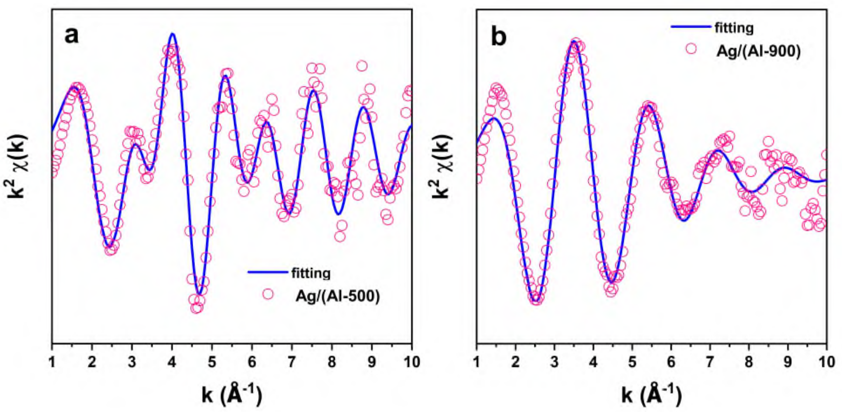

Supplementary Figure 9. EXAFS fitting curve in k space. **a** (Ag/Al-500). **b** Ag/(Al-900).

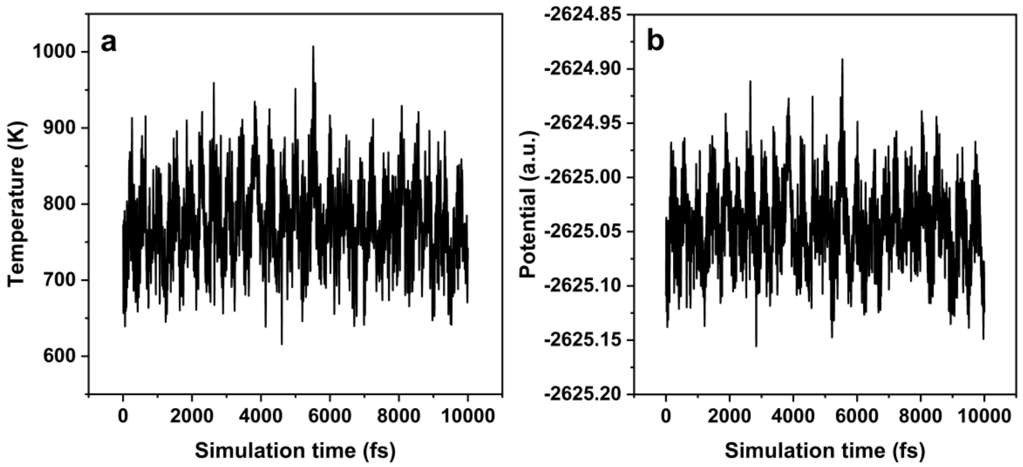

Supplementary Figure 10. Temperature and potential energy diagrams for AIMD.

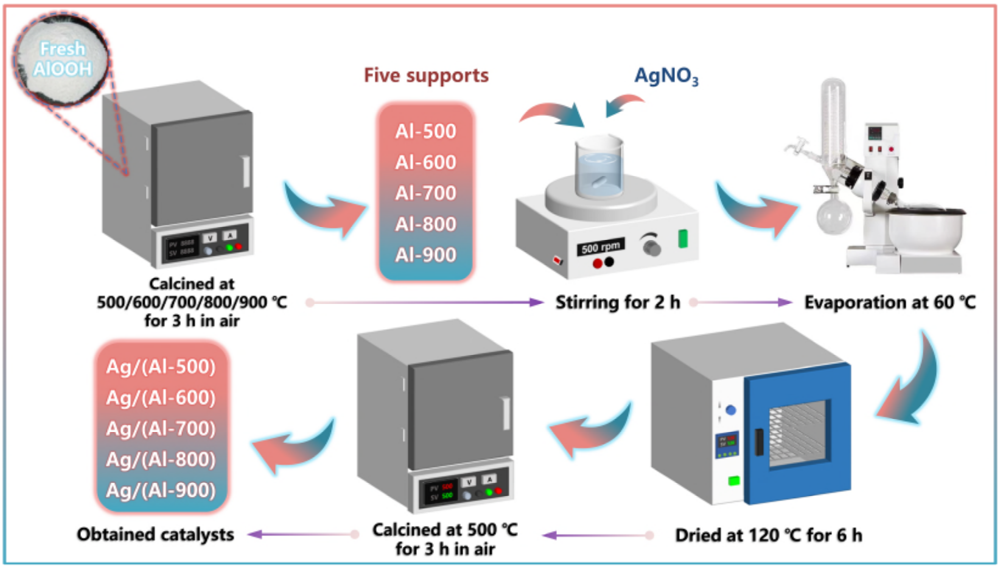

Supplementary Figure 11. Catalyst synthesis route.

Supplementary Table 1. BET surface area, pore size and Pore volumes of the Ag/(Al-X) catalysts calcined at different temperatures. (X=500 °C, 600 °C, 700 °C, 800 °C, 900 °C)

Sample	S_{BET} (m^2 g^{-1})	Pore diameter (d) (nm)	Pore volume (V) (cm^3 g^{-1})
Ag/(Al-500)	325.5	3.9	0.72
Ag/(Al-600)	289.7	6.6	0.74
Ag/(Al-700)	255.4	6.6	0.73
Ag/(Al-800)	231.3	7.8	0.69
Ag/(Al-900)	182.4	7.9	0.55

Supplementary Table 2. Three hydroxyl areas of AlOOH calcined at different temperatures.

Sample	Peak 1 (3790)	Peak 2 (3741)	Peak 3 (3675)	Total area
Al-500	0.42	6.69	4.35	11.5
Al-600	0.65	4.86	3.30	8.4
Al-700	0.87	5.85	3.27	10
Al-800	1.13	5.68	3.32	10.2
Al-900	1.19	5.29	3.22	9.6

Supplementary Table 3. EXAFS fitting parameters at the Ag K-edge for various samples

Sample	Shell	*CN*[a]	*R*(Å)[b]	σ^2(Å^2)[c]	ΔE_0(eV)[d]	R factor
Ag foil	Ag-Ag	12	2.86	0.009	0.3±0.1	0.002
Ag/(Al-500)	Ag-O	2.9±0.1	2.25	0.024	-2.0±0.2	0.007
	Ag-Ag	3.6±0.1	2.86	0.014		
Ag/(Al-900)	Ag-O	3.8±0.1	2.29	0.017	-1.9±0.1	0.001

[a] *CN*, coordination number;

[b] *R*, the distance to the neighboring atom;

[c] σ^2, the Mean Square Relative Displacement (MSRD);

[d] ΔE_0, inner potential correction.
